# Dietary Intake and Asthma in Preschoolers: A Logistic Lasso Regression Analysis

**DOI:** 10.3389/fped.2022.870529

**Published:** 2022-06-03

**Authors:** Yangming Qu, Chengliang Pan, Shijie Guo, Hui Wu

**Affiliations:** ^1^Department of Neonatology, The First Hospital of Jilin University, Changchun, China; ^2^College Clinical Medicine, Jilin University, Changchun, China

**Keywords:** asthma, dietary intake, lasso regression, child, health

## Abstract

**Background:**

Asthma is a common chronic disease among children, especially preschoolers. Some evidence suggests that diet may play a role in asthma, but the current findings are contradictory. The objective of our study was to determine the association between dietary intake and asthma in preschool children aged 2–5 years.

**Methods:**

We selected preschool children aged 2–5 years with complete data on asthma diagnosis, diet, and body mass index (BMI) from the national health and nutrition examination survey (NHANES) database. In a selected population, children with self-reported asthma were included in the final sample. In children without self-reported asthma, we further used propensity score matching (PSM) to match age and sex for sampling, maintaining a ratio of 1:4 for cases. Lasso regression was used to identify dietary factors affecting asthma in preschoolers.

**Results:**

A total of 269 children with self-reported asthma and 1,076 children without self-reported asthma were included in our study. Univariate analysis showed that there were significant differences in ethnicity and dietary zinc intake between asthmatic children and children without asthma. After adjusting for all dietary and demographic variables, the results of logistic Lasso regression analysis showed that non-Hispanic black (β = 0.65), vitamin B12 (β = 0.14), and sodium (β = 0.05) were positively associated with childhood asthma, while Vitamin K (β = −0.04) was negatively associated with childhood asthma.

**Conclusion:**

In conclusion, our study confirms that non-Hispanic black and dietary sodium intake are associated with a higher risk of asthma in preschoolers. In addition, our study found that dietary vitamin B12 was positively associated with childhood asthma, while vitamin K was negatively associated with childhood asthma.

## Introduction

Asthma is a common chronic disease in children, characterized by chronic inflammation, airway hyper responsiveness, and periodic airflow obstruction (1). According to the National Center for Health Statistics, the prevalence of asthma in children under 18 years used to reach 13% and remained at 8.4% as of 2017 (2). Preschoolers are more susceptible, with higher rates of asthma prevalence, attacks, visits, and hospitalizations (3). In Sweden, the prevalence of asthma in preschool children is about 9% (4, 5). In Portugal, at least one out of every three to four preschoolers have an asthma episode (6).

The main symptoms of childhood asthma include wheezing, coughing, chest tightness, and choking. While most of the symptoms in children can be controlled by inhaling glucocorticoids, some children experience severe asthma attacks, decreased lung function, and death, even with high doses of glucocorticoids (7). Besides, asthma can also cause mental health problems in children. Previous studies have shown that children with asthma are more likely to have neurodevelopmental, behavioral and emotional problems, and learning disabilities than their healthy peers (8–11).

The established risk factors for childhood asthma include genes, environment, and obesity (12). Due to the immutability of gene and the diversity of allergens of asthma, it is difficult to prevent childhood asthma from a genetic or environmental perspective. As a major factor in controlling obesity, some evidence suggests that diet may play a role in asthma (13, 14). However, most studies have focused on dietary patterns or single nutrients, with conflicting results, and not enough data to assess the impact of asthma on children. In addition, the possible multicollinearity between dietary macronutrients and micronutrients poses challenges to statistical techniques.

National health and nutrition examination survey is a study conducted by the National Center for Health Statistics of the Centers for Disease Control and Prevention (CDC) to assess the health and nutritional status of the U.S. population. Data from 5,000 participants have been collected annually since 1999. Lasso is a special modern statistical technique that allows a large number of covariables in the model, and can actively select risk factors from a set of potentially multicollinearity variables, resulting in a more relevant and explainable set of predictors (15). Our study combined high-quality data with modern statistical techniques to analyze dietary risk factors for asthma in preschoolers aged 2–5 years.

## Methods

### Study Design

**Figure 1:**
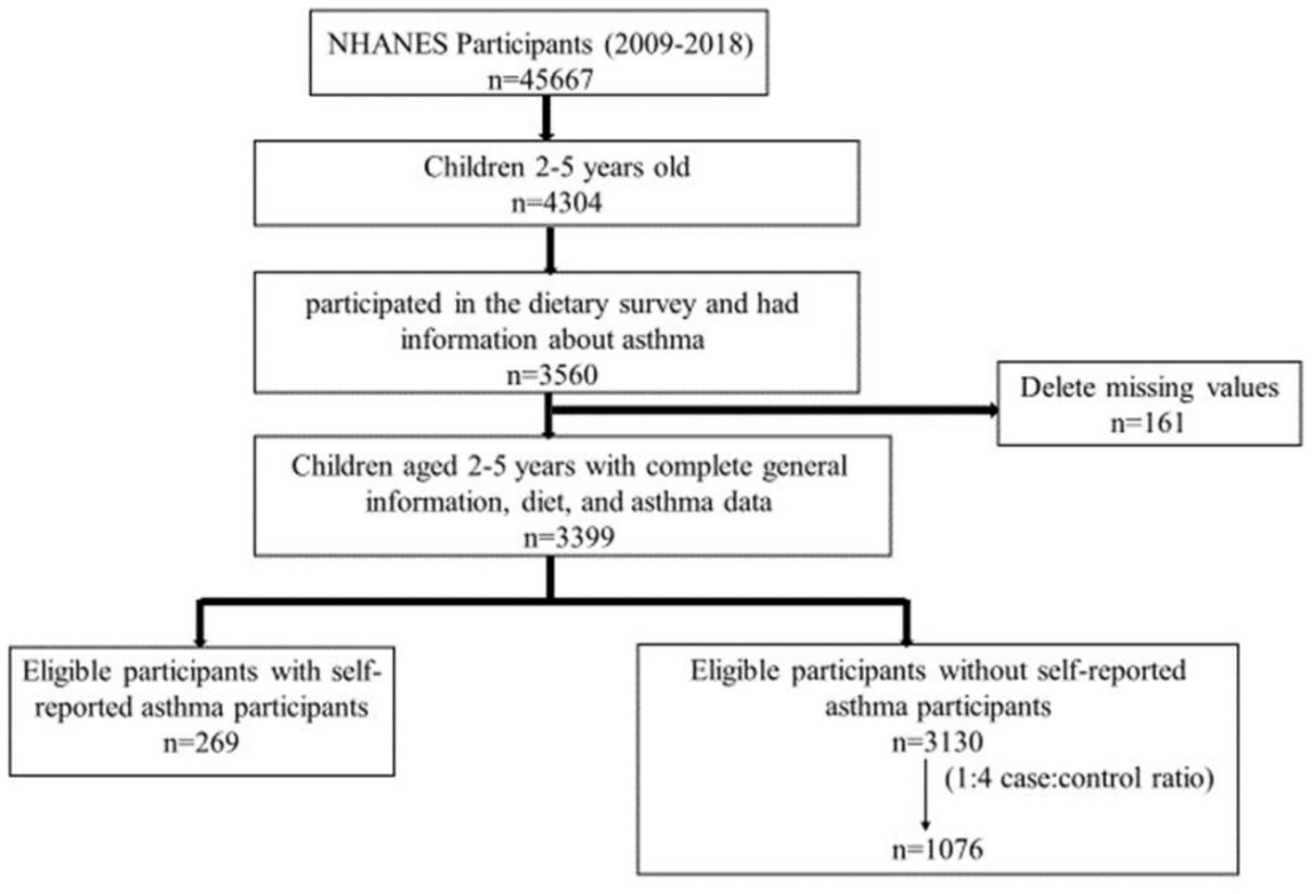
Flowchart of study participants.

Our study is a case-control study. The NHANES collected information on demographic, socioeconomic, and health-related factors, as well as a 24-h dietary recall assessment. From 2011 to 2016, the NHANES included 45,667 participants, including 4,304 children aged 2–5 years. Among all the children aged 2–5 years, 3,560 participants participated in the dietary survey and had information about asthma. After deleting the missing values, we selected 3,399 preschoolers aged 2–5 years with complete demographic information, diet, and asthma data. The selected population can well represent the 2–5 years old population in NHANES (Supplementary Tables 1, 2). In the selected population, 269 children with self-reported asthma were included in the final sample. For better statistical power, among the children without self-reported asthma in the selected population (*n* = 3,130), we further used propensity score matching (PSM) to match age and sex for sampling, maintaining a ratio of 1:4 for cases, resulting in a final study sample of children with self-reported asthma (*n* = 269) and children without self-reported asthma (*n* = 1,076) (Figure 1). Since NHANES is a publicly available dataset, the current study is exempt from approval by an Institutional Review Board. All participants provided informed consent.

### Information of Asthma

Data on self-reported asthma was recorded in the “Medical Conditions” section of the NHANES interview. A proxy answered the following questions for children under 16 years of age in NHANES: “Has a doctor or other health professional ever told that the survey participant has asthma?” followed by “Does the survey participant still have asthma?” Only children who still had asthma at the time of the survey were included in the case group.

### Dietary Intake

Dietary intakes were obtained from the “Total Nutrient Intake” section of the NHANSE dietary interview. USDA Food and Nutrient Database for Dietary Studies 5.0 (FNDDS 5.0) was used to estimate the intake of energy, nutrients, and other food components in foods and beverages consumed by the participants during the 24 h prior to the interview (from midnight to midnight).

### Other Measures

Age, gender, and ethnicity were obtained from the demographic variables and sample weights dataset. The information of household smoker was obtained from household smoker dataset. Body mass index (BMI) of the participants was obtained from the Body Measures dataset.

### Statistical Analysis

All statistical analyses were performed using R Statistical Software (version 4.0.3). Due to the stratified, multi-stage probabilistic cluster sampling design of NHANES, the “survey” package was used in the univariate analyses. We included five cycles of NHANES data from 2009 to 2018, and adjusted the data for 2-year dietary weight. The stratification variable (SDMVSTRA) and primary sampling unit variable (SDMVPSU) were selected according to the study design to appropriately adjust the variance estimates. The dietary variables were log-transformed appropriately, statistical analysis (including univariate test and Lasso regression) was performed on the log-transformed data, and statistical description was performed on the raw/non-log transformed data. For statistical description, the $\bar{\chi }\pm$S was used for normal continuous variables, the Q (*P*_25_-*P*_75_) was used for non-normal continuous variables, and *n* (%) was used for discrete data. For univariate analysis, the *t*-test was performed for normal continuous variables, rank sum test was performed for no-normal continuous variables, and chi-square test was performed for discrete variables. A *p*-value < 0.05 (two-tailed) was considered to be statistically significant.

**Figure 2:**
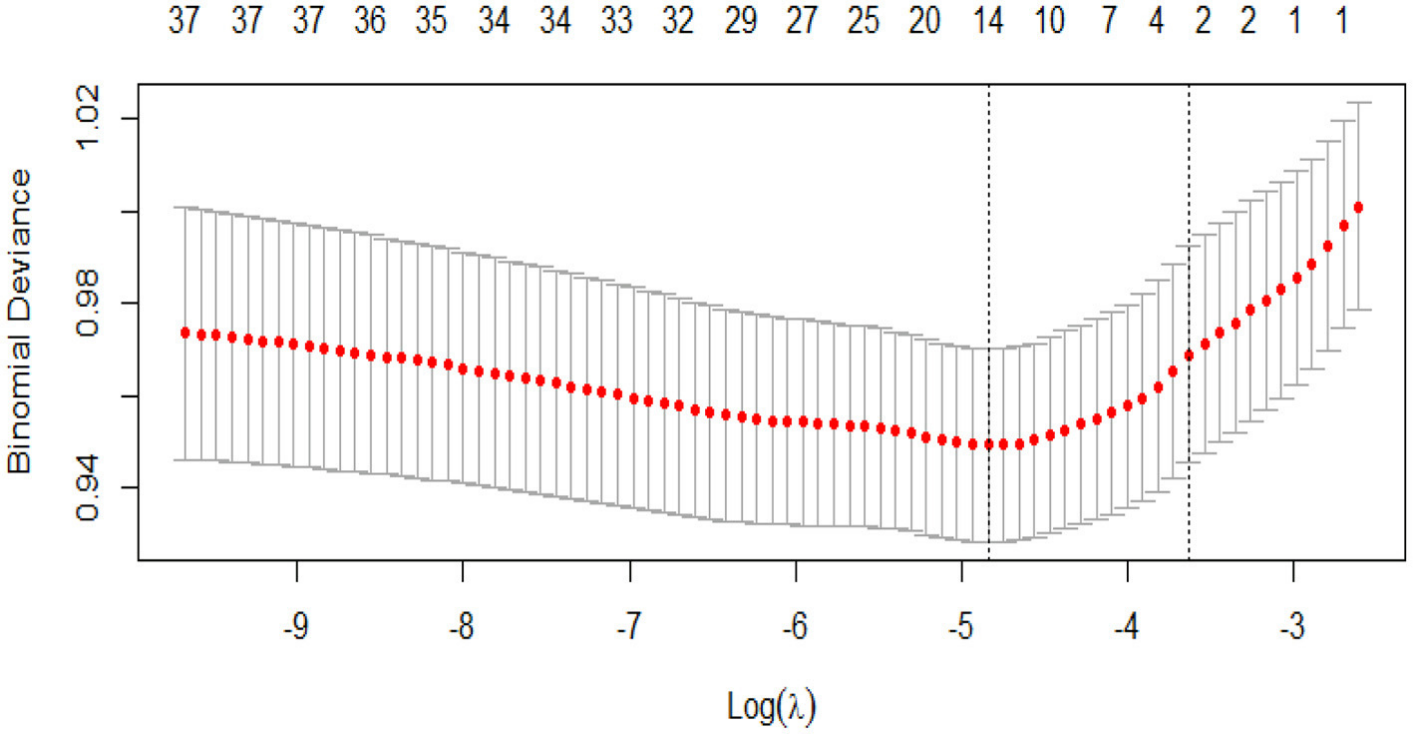
Cross-validation plot for the penalty term.

The “glmnet” package was used to fit the logistic Lasso regression. Asthma was included in the logistic LASSO regression as the dependent variable Y, coded 0 represents children without asthma, 1 represents children without asthma. We included 31 dietary variables as continuous variables into the model. In addition, five demographic variables (including gender, age, household smoker, ethnicity, and BMI) were also included in the logistic Lasso model. Ten-fold cross-validation was used to select the penalty term lambda (λ). Binomial deviation was used to measure the prediction performance of the fitting model. The built-in function in R produces two automatic λ's, and we chose lambda.1se (the largest λ within one standard error range of the minimal binomial deviation) because it results in a stricter penalty and a smaller covariable number than lambda.min (λ with the minimal binomial deviation). In our study, lambda.min was 0.0079 and lambda.1se was 0.0265 (Figure 2).

## Results

### Demographic Characteristics

There was significant difference in ethnicity between children with and without asthma (*p* < 0.05). There was no significant difference in gender, age, household smoker, and BMI between children with and without asthma (*p* > 0.05). The demographic characteristics of the study population are shown in Table 1.

**Table 1:** The demographic characteristics of the study population.

**Demographic variable**	**Children without asthma** **(*n* = 1,076)**	**Children with asthma** **(*n* = 269)**	***p*-value**
Gender, *n*, (%)			1.000
Male	680 (63.2%)	170 (63.2%)	
Female	396 (36.8%)	99 (36.8%)	
Age, mean ± SD	3.55 ± 1.13	3.55 ± 1.13	1.000
Household smoker, *n*, (%)			0.593
No	764 (71.0%)	198 (73.6%)	
Yes	312 (29.0%)	71 (26.4%)	
Ethnicity, *n*, (%)			<0.001
Mexican American	192 (17.8%)	30 (11.2%)	
Other Hispanic	95 (8.8%)	22 (8.2%)	
Non-Hispanic white	378 (35.1%)	67 (24.9%)	
Non-Hispanic black	225 (20.9%)	110 (40.9%)	
Other race	186 (17.4%)	40 (14.8%)	
BMI	16.50 ± 1.92	16.74 ± 2.26	0.118

### Dietary Intake

The dietary intake of children with and without asthma is shown in Table 2. Univariate analysis showed significant difference in dietary zinc intake between children with asthma and children without asthma [7.72 (5.51–11.12) vs. 7.07 (4.97–9.66); *p* = 0.015]. There was no significant difference in other dietary variables between children with and without asthma (*p* > 0.05).

**Table 2:** Dietary intake of children with and without asthma.

**Dietary variables**	**Children without asthma** **(*n* = 1,076)**	**Children with asthma** **(*n* = 269)**	***p*-value**
Energy (Kcal)	1,449 (1,138–1,816)	1,509 (1,203–1,917)	0.173
Protein	49.82 (36.89–65.09)	52.69 (40.38–71.50)	0.181
Protein, % energy	13.73 (11.57–16.01)	13.46 (11.30–16.58)	0.829
Carbohydrate	193.80 (150.32–246.54)	205.08 (156.29–271.21)	0.199
Carbohydrate, % energy	54.02 (48.78–59.61)	53.49 (47.70–59.78)	0.698
Fat	52.74 (39.29–70.83)	55.14 (40.95–76.55)	0.255
Fat, % energy	33.42 (28.31–37.79)	33.45 (28.08–38.43)	0.979
Cholesterol (mg)	139.50 (83.75–229.25)	149.00 (99.00–236.00)	0.458
Fiber (g)	11.00 (7.58–15.30)	10.20 (7.40–14.70)	0.423
Folate (μg)	254.50 (182.00–366.00)	275.00 (189.00–373.00)	0.089
Vitamin B12 (μg)	3.42 (2.17–5.01)	3.97 (2.66–5.80)	0.110
Vitamin B6 (mg)	1.31 (0.92–1.78)	1.41 (1.03–1.90)	0.089
Thiamin (Vitamin B1) (mg)	1.17 (0.86–1.56)	1.23 (0.87–1.69)	0.119
Riboflavin (Vitamin B2) (mg)	1.52 (1.09–2.01)	1.58 (1.20–2.24)	0.103
Vitamin B3 (mg)	15.20 (10.71–20.10)	11.49 (11.49–21.98)	0.060
Choline (μg)	188.35 (134.00–259.35)	201.70 (142.80–272.50)	0.403
Calcium (mg)	824.00 (548.00–1,156.25)	876.00 (566.00–1,272.00)	0.100
Phosphorous (mg)	987.00 (748.00–1131.00)	1,028.00 (755.00–1,369.00)	0.154
Magnesium (mg)	186.00 (144.00–244.00)	187.00 (144.00–246.00)	0.373
Iron (mg)	9.78 (7.00–13.85)	10.63 (7.36–14.72)	0.171
Vitamin A (RE)	458.00 (286.75–694.00)	509.00 (320.00–723.00)	0.265
Vitamin C (mg)	62.00 (28.20–109.13)	71.80 (32.30–117.90)	0.378
Vitamin D (μg)	4.70 (2.60–7.40)	5.30 (3.00–8.00)	0.328
Vitamin E (mg)	5.11 (3.46–7.44)	4.61 (3.13–7.25)	0.553
Vitamin K (μg)	38.20 (24.38–60.95)	33.10 (20.20–54.00)	0.242
Zinc (mg)	7.07 (4.97–9.66)	7.72 (5.51–11.12)	0.015
Sodium (mg)	2,068.50 (1,549.00–2,741.00)	2,330.00 (1,680.00–3,083.00)	0.126
Potassium (mg)	1,832.00 (1,380.50–2,416.00)	1,910.00 (1,440.00–2,403.00)	0.304
Selenium (μg)	67.00 (47.48–89.35)	69.50 (50.70–96.50)	0.259
Theobromine(g)	6.00 (0–46.00)	3.00 (0–55.00)	0.706
Caffeine (mg)	1.00 (0–6.00)	1.00 (0–7.00)	0.532

### Lasso Regression Analysis

**Figure 3:**
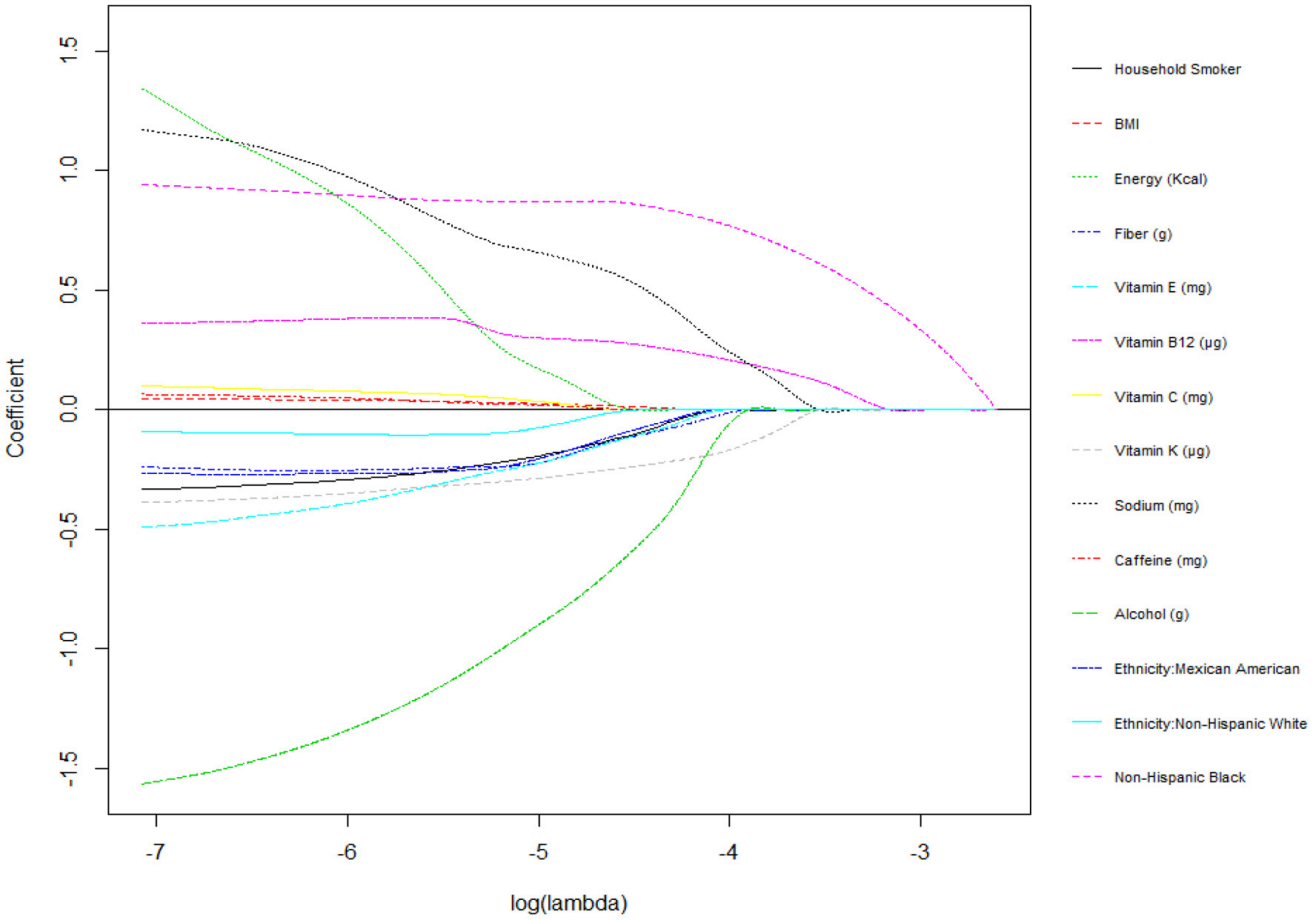
Plots for Lasso regression coefficients.

Figure 3 and Table 3 show the coefficient of variables in Lasso regression at λ = 0.0265. After adjusting for all dietary and demographic variables, the results of logistic Lasso regression analysis showed that non-Hispanic black (β = 0.65), vitamin B12 (β = 0.14), and sodium (β = 0.05) were positively associated with childhood asthma, while vitamin K (β = −0.04) was negatively associated with childhood asthma.

**Table 3:** The estimated coefficients for logistic Lasso regression.

**Variables**	**Coefficients**
Demographic variable	
Gender	0
Age (years)	0
Mexican American	0
Other Hispanic	0
Non-Hispanic White	0
Non-Hispanic Black	0.65
Other race	0
Household smoker	0
BMI	0
Dietary variables	
Energy (Kcal)	0
Carbohydrate, % energy	0
Protein, % energy	0
Fat, % energy	0
Cholesterol (mg)	0
Fiber (g)	0
Folate (μg)	0
Vitamin B12 (μg)	0.14
Vitamin B6 (mg)	0
Thiamin (Vitamin B1) (mg)	0
Riboflavin (Vitamin B2) (mg)	0
Vitamin B3(mg)	0
Choline (μg)	0
Calcium (mg)	0
Phosphorous (mg)	0
Magnesium (mg)	0
Iron (mg)	0
Vitamin A (RE)	0
Vitamin C (mg)	0
Vitamin D (μg)	0
Vitamin E (mg)	0
Vitamin K (μg)	−0.04
Zinc (mg)	0
Sodium (mg)	0.05
Potassium (mg)	0
Selenium (μg)	0
Alcohol (g)	0
Theobromine(g)	0
Caffeine (mg)	0

## Discussion

To our knowledge, this is the first study to assess the relationship between multitude of dietary variables and the risk of asthma using the powerful Lasso shrinkage technique. The logistic Lasso model is a regression-based shrinkage method that can actively select a set of more relevant and interpretable predictors from a large, potentially multicollinear set of variables regression (16). In this method, continuous shrinkage operation is used to reduce the sum of the absolute values of the regression coefficients to reduce the possibility of over fitting. As shrinkage increases, some coefficients even reach 0, thus automatically removing unnecessary/non-influential covariates and leaving non-zero variables in the model. To date, most studies of the relationship between diet and disease have used traditional statistical techniques. Since traditional regression techniques are limited in analyzing and synthesizing a large number of covariables (including multicollinearity variables), only a few dietary variables can be included or only dietary patterns can be analyzed. With the availability of large amounts of dietary and health data, combined with advanced statistical techniques, we have a new opportunity to elucidate novel associations, patterns, and clusters not previously observed with traditional statistical methods, which may contribute to and provide a more robust understanding of the role of diet in childhood asthma.

We used data from a large national nutrition survey combined with a robust Lasso regression technique to minimize multicollinearity between dietary variables and obtain the true associations between dietary intake and asthma in preschool children. Results from our initial univariate analysis suggest that ethnicity and dietary zinc are associated with childhood asthma. The results of the ultimate Lasso regression analysis showed that non-Hispanic black children had a higher risk of asthma. In addition, dietary vitamin B12 and sodium were positively associated with childhood asthma, while vitamin K was negatively associated with childhood asthma.

Many studies have analyzed the association between childhood asthma and ethnicity. Overall, studies showed that non-Hispanic children were at greater risk for asthma than Hispanic children (17–19). Phuong et al. (20) further divided non-Hispanic into non-Hispanic white and non-Hispanic black in a larger cross-sectional study and found that non-Hispanic black was associated with an increased risk of childhood asthma. Corinne et al. (21) also showed that non-Hispanic black was an independent risk factor for childhood asthma and the increased risk of asthma in non-Hispanic black children may be associated with economic status. Our findings are consistent with previous studies.

Dietary sodium was considered to increase the risk of asthma. Morbidity and mortality of asthma were observed to be higher in communities with more Westernized lifestyles (typically with higher dietary sodium intake) and among migrants from underdeveloped rural to Westernized urban areas (22, 23). Epidemiological studies have found that children are at greater risk for sodium-related asthma because age is an important factor in determining sodium sensitivity (24). Our study confirmed that dietary sodium increases the risk of asthma in children. In addition, intervention studies have also shown that a low-sodium diet is associated with reduced asthma severity (24).

Our study also found a positive association between dietary vitamin B12 and childhood asthma, which is inconsistent with the findings of current epidemiological studies (25, 26). However, it is theorized that vitamin B12 may influence asthma risk by affecting DNA methylation. DNA methylation is an epigenetic regulation that may influence the pathogenesis of asthma by increasing or decreasing the expression level of susceptibility genes (27). Most methyl donors for DNA methylation in humans derived from methyl groups in the diet (including and vitamin B12). In a mouse model, the severity of allergic airway disease (AAD) was increased in the F1 (offspring) and F2 generations of maternal rats whose diets were rich in methyl donors (including folic acid, vitamin B12, and choline) (28). Based on previous animal and clinical studies, choline's effects are more likely to reduce (rather than increase) airway inflammation (29–31). Therefore, folic acid and vitamin B12 are highly likely to increase the risk of AAD. The true association between vitamin B12 and asthma is uncertain because there is no animal model for the effects of vitamin B12 or folic acid on experimental asthma (AAD).

In recent years, the role of fat-soluble vitamins (D, E, K, and A) in asthma has attracted extensive attention. Several studies (32, 33) have analyzed the role of vitamins A, D, and E in asthma, but there is little evidence of the role of vitamin K in asthma. Our study found that dietary vitamin K was associated with a reduced risk of asthma in children. The protective effect of vitamin K on asthma may depend on vitamin K-dependent protein (VKdP). VKdPs are a group of proteins that require vitamin K to conduct carboxylation. Low vitamin K intake may lead to a decrease in the carboxylation of VKdPs (34, 35). At present, a total of 17 VKdPs have been identified in the human body, and these VKdPs play an important role in a variety of diseases, among which periosteal and Gas6 have been found to be beneficial to asthma patients (36, 37). The periosteal can increase the adhesion of eosinophils to fibronectin, activate TGF-β-mediated (transforming growth factor-β-mediated) fibroblasts to increase the generation of type I collagen, and participate in the process of sub epithelial fibrosis through its fibrogenic function, promoting airway remodeling in asthma (38–41). Gas6 is an effective modulator of lung remodeling responses because of its direct effect on various cellular components of airways and blood vessels (37). In addition, vitamin K's powerful ability to inhibit the release of inflammatory cytokines may also be beneficial for asthma (42). Our study found that other fat-soluble vitamins (D, E, and A) are not significantly associated with asthma, which may be related to the collinearity between nutrients.

The advantages of our study included the following: (1) NHANES database has collected a large number of national samples, which has sufficient research efficiency and extensibility; (2) NHANES oversampled different racial groups, such as Hispanics and African-Americans, which could compensate for the underrepresentation of groups in previous studies on diet and asthma; (3) modern statistical techniques were applied to our study to solve the problem of collinearity between micronutrients and macronutrients. There are some limitations to our study: (1) this is a case-control study with self-reported diet and asthma data; (2) patients may have changed their diet after diagnosis, which may have influenced the results; (3) the number of asthma-related variables included in our study remains small.

## Conclusion

In conclusion, our study confirms that non-Hispanic black and dietary sodium intake are associated with a higher risk of asthma in preschoolers. In addition, our study found that dietary vitamin B12 was positively associated with childhood asthma, while vitamin K was negatively associated with childhood asthma.

## Data Availability Statement

Publicly available datasets were analyzed in this study. This data can be found at: https://www.cdc.gov/nchs/nhanes/index.html.

## Ethics Statement

Ethical approval was not provided for this study on human participants because we used data from NHANES database. Since NHANES is a publicly available dataset, the current study is exempt from approval by an Institutional Review Board. Written informed consent to participate in this study was provided by the participants' legal guardian/next of kin.

## Author Contributions

HW and YQ conceptualized and designed the study, drafted the initial manuscript, and reviewed and revised the manuscript. YQ, CP, and SG collected data, carried out the initial analyses, and reviewed and revised the manuscript. All authors approved the final manuscript as submitted and agree to be accountable for all aspects of the work.

## Conflict of Interest

The authors declare that the research was conducted in the absence of any commercial or financial relationships that could be construed as a potential conflict of interest.

## Publisher's Note

All claims expressed in this article are solely those of the authors and do not necessarily represent those of their affiliated organizations, or those of the publisher, the editors and the reviewers. Any product that may be evaluated in this article, or claim that may be made by its manufacturer, is not guaranteed or endorsed by the publisher.
