# Supplementary material for: Dietary Intake and Asthma in Preschoolers: A Logistic Lasso Regression Analysis
Source: Front Pediatr. 2022 Jun 3;10:870529. doi: 10.3389/fped.2022.870529 (PMC9204041; doi:10.3389/fped.2022.870529)
Supplement: Supplementary file 1 [file Table_1.DOCX]

Supplementary table 1 comparison of the characteristics of 3399 selected children with children with asthma and diet data

| Demographic variable | Population 1(n=3399) | Population 2 (n=3560) | *p* |
| --- | --- | --- | --- |
| Gender, n, (%) |  |  | 0.919 |
| Male | 1702(50.1%) | 1788(50.0%) |  |
| female | 1697(49.9%) | 1772(50.0%) |  |
| age, mean ± SD | 3.34±1.14 | 3.34±1.14 | 0.954 |
| Ethnicity, n, (%) |  |  | 0.983 |
| Mexican American | 720(21.2%) | 770(21.6%) |  |
| Other Hispanic | 356(10.5%) | 371(10.4%) |  |
| Non-Hispanic White | 1005(29.6%) | 1061(29.8%) |  |
| Non-Hispanic Black | 800(23.5%) | 828(23.3%) |  |
| Other Race | 518(15.2%) | 530(14.9%) |  |
| BMI | 16.47±1.14 | 16.47±1.14 | 0.977 |
| Asthma |  |  | 0.903 |
| No | 3128(92.0%) | 3280(92.1%) |  |
| Yes | 271(8.0%) | 280(7.9%) |  |

* Population 1: 3399 preschoolers aged 2-5 years with complete general information, diet, and asthma data(the missing value has been deleted); Population 2: 3,560 participants aged 2-5 years participated in the dietary and asthma survey

Supplementary table 2 comparison of the characteristics of 3399 selected children with all 2-5 years of age in the NHANES

| Demographic variable | Population 1 (n=3399) | Population 3 (n=4304) | *p* |
| --- | --- | --- | --- |
| Gender, n, (%) |  |  | 0.433 |
| Male | 1702(50.1%) | 2195(51.0%) |  |
| female | 1697(49.9%) | 2109(49.0%) |  |
| age, mean ± SD | 3.34±1.14 | 3.35±1.14 | 0.527 |
| Ethnicity, n, (%) |  |  | 0.806 |
| Mexican American | 720(21.2%) | 889(20.7%) |  |
| Other Hispanic | 356(10.5%) | 462(10.7%) |  |
| Non-Hispanic White | 1005(29.6%) | 1233(28.6%) |  |
| Non-Hispanic Black | 800(23.5%) | 1041(24.2%) |  |
| Other Race | 518(15.2%) | 679(15.8%) |  |
| BMI | 16.47±1.14 | 16.45±1.14 | 0.737 |
| Asthma |  |  | 0.063 |
| No | 3128(92.0%) | 3956(91.9%) |  |
| Yes | 271(8.0%) | 341(7.9%) |  |
| NA | 0(0) | 7(0.2%) |  |

* Population 1: 3399 preschoolers aged 2-5 years with complete general information, diet, and asthma data(the missing value has been deleted); Population 3: all children aged 2-5 years participated in NHANES
